# Human health and ecological risk assessment of heavy metals in topsoil of different peatland use types

**DOI:** 10.1016/j.heliyon.2024.e33624

**Published:** 2024-06-25

**Authors:** Samuel Obeng Apori, Michelle Giltrap, Julie Dunne, Furong Tian

**Affiliations:** a School of Food Science and Environmental Health, Technological University Dublin, City Campus, Grangegorman, D07ADY7, Dublin, Ireland; b Nanolab Research Centre, Physical to Life Sciences Hub, Technological University Dublin, D08 CKP1, 11 Dublin, Ireland; c FOCAS Research Institute, Radiation and Environmental Science Centre, Technological University Dublin, City Campus, Camden Row, D08C KP1, 11 Dublin, Ireland

**Keywords:** Peatlands, Heavy metals, Land use changes and risk assessment

## Abstract

Peatlands, known for their ability to retain and immobilize heavy metals due to unique waterlogged conditions and organic matter, face challenges when subjected to disturbances such as land use changes. These disruptions alter the organic matter, redox potential, and pH of the peatsoil, potentially influencing the migration, mobilization, and increased availability of stored heavy metals. Peatsoil samples from various peatland use types (improved and semi-natural grassland, forest, industrial cutaway bog) were collected to assess the human health and ecological risk associated with heavy metals (Cd, Cu, Hg, Pb, and Zn) in Co-Offaly, Ireland. Results reveal variations in heavy metal concentrations across peatland use types, with Cd, Hg, and Pb in improved and semi-natural grassland peatsoils exceeding the World Health Organization (WHO) permissible safety limits. Contamination factors (CF) were higher in improved grassland, especially for Cd and Pb, exceeding one. Hakanson potential ecological risk assessment indicates acceptable overall risk levels, though variations exist between improved grassland, unimproved grassland, forest, and industrial cutaway bog. Combined exposure routes (dermal, ingestion and inhalation routes) to all heavy metals do not exceed safe exposure levels (indicating low non-carcinogenic risks. However, the cancer risk (CR) exceeds acceptable thresholds across all use types, with higher CR in improved grassland, especially for children. Overall, the findings emphasize the need for careful consideration of heavy metal risks associated with land use changes in peatlands, particularly in the improved grassland areas.

## 1 Introduction

Peatlands, with their unique waterlogged conditions and organic matter, are effective at retaining and immobilizing heavy metals [1]. Numerous studies have confirmed the capacity of peatlands to function as sinks for potentially toxic elements (i.e including zinc (Zn), copper (Cu), lead (Pb), mercury (Hg) and cadmium (Cd)) [2]. For instance, Headley (1996) and Borgulat et al. (2018) both found high concentrations of heavy metals (Cu, Zn, Ni and Fe) in peat exceeding the World Health Organization (WHO) permissible thresholds [[2], [3]]. The former identified soil as the ultimate sink for heavy metal accumulation and the latter demonstrated the role of peat in retaining different metals. This work was further supported by Syrovetnik et al. [4]. However, disturbances such as those caused by land use changes can alter the redox potential and pH of the soil, potentially releasing these stored metals [5]. The release of metals can be exacerbated by drainage, which lowers the water tables and releases acidic, metal-contaminated waters from peatlands [5].

As peatlands are drained to make them suitable for agricultural and other land uses, it not only alters the hydrological dynamics of the ecosystem [6] but also impacts soil health [7,8]. The drainage process accelerated the decomposition of organic matter, causing a decline in soil organic content [9]. Peatlands, characterized by naturally acidic conditions, undergo a shift towards neutral levels as a result of drainage and decomposition processes. The rise in pH, coupled with reduced organic matter, creates an environment conducive to the mobilization and availability of the stored heavy metals in peatland. Li et al. [10], found a significant positive correlation between soil organic matter content and heavy metal content, while soil acidity was negatively correlated with some heavy metals. This was further supported by B. Li et al. [11], as they highlighted the organic matter in controlling heavy metal pollution. Therefore, metal ions which were once bound to organic matter, are released into the soil solution, posing a potential threat to both human health and the broader ecological balance.

Peatlands were once regarded as protective environments with limited human interaction. However, upon conversion to other land uses, the interaction between altered peatlands and humans increases, potentially exposing humans to the available heavy metals found in peatsoils. Human exposure to heavy metals, such as cadmium, lead, mercury, and copper, poses significant health risks [[12], [13], [14], [15], [16]]. This exposure can occur through various pathways, including ingestion, inhalation, and dermal contact [[17], [18], [19], [20]]. The health hazards associated with heavy metal exposure include neurotoxicity, carcinogenicity, and osteoporosis [16,19,21], with those living near or working on forested peatland, industrial cutaway bog and peatland converted for improved grassland will be at a higher risk of exposure.

A range of studies have highlighted the significant health risks posed by heavy metal contamination in different ecosystems [17,[22], [23], [24], [25]]. In the Pearl River Delta urban agglomeration of China, Zheng et al. [26] found that cadmium was the most common pollutant, with the highest risks associated with rice and maize consumption. Similarly, Hu et al. [27] identified high potential carcinogenic risk in children due to arsenic and lead in rapidly developing regions of China. In the Klang district of Malaysia, Yuswir et al. [28], found that heavy metal contamination was below acceptable thresholds for adults but exceeded them for children. In China, Yang et al. [29], reported health risks from heavy metal exposure in farmland, with arsenic, chromium, and cadmium posing the greatest concerns. X. Liu et al. [30], found significant heavy metal contamination in soils, with chromium and lead posing the primary non-cancer risks and cadmium the greatest cancer risk while the heavy metal contents (Cr, Pb, Zn, Cu, Ni) in soils and sand from playgrounds in Çanakkale, Turkey were below international limits, indicating no immediate need for remediation [24]. While numerous studies have addressed heavy metal contamination in various ecosystems, none have specifically focused on human health risk assessment associated with potentially toxic elements in altered peatlands, despite their recognized role as heavy metal sinks.

In aligning our research with Sustainable Development Goal #3 (SDG3), which aims to ensure good health and well-being for all, we employ a comprehensive and interconnected approach, integrating soil science, ecology, and public health disciplines [31]. Therefore, this research seeks to investigate the heavy metal content in topsoils under various peatland use types—grassland (both improved and semi-natural), forestry, and industrial cutaway—aiming to provide insights into the mechanisms underlying the intricate relationships between land use change, heavy metal availability, and potential ecological and human health risks under different peatland use types. The results of this study can provide valuable insights for implementing sustainable land management strategies, mitigating environmental risks, and contributing to the establishment of guidelines for peatland uses that prioritise the well-being of humans and the integrity of the ecosystem.

## 2 Methodology

### 2.1 Study area

**Fig. 1:**
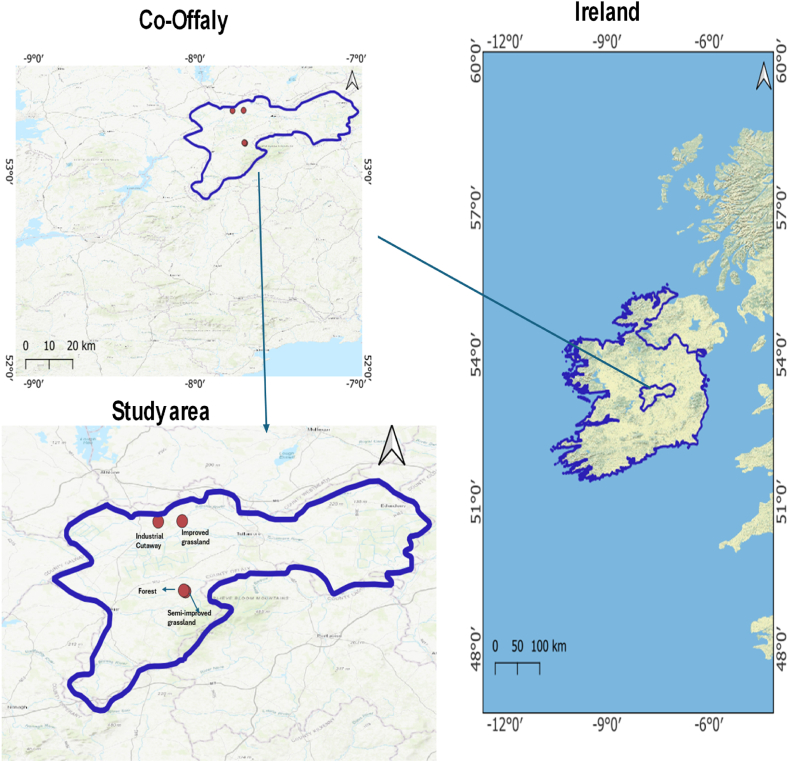
Location of the study area in the Republic of Ireland.

The research was carried out in improved grassland (53°18′46.9″N 7°41′53.4″W), semi-natural grassland (53°08′52.6″N 7°41′37.0″W), industrial cutaway (53°18′42.2″N 7°46′00.9″W), and forestry (53°08′44.6″N 7°41′18.8″W) at Co-Offaly located in the midlands of the Republic of Ireland (Fig. 1).

The annual air temperature in the county exhibits a range of 5.7 °C–13.0 °C, with a mean precipitation of 819 mm [32]. The peatlands of County Offaly, Ireland, are a significant part of the country's landscape, covering approximately 20.6 % of the national land area [33]. These peatlands are a valuable resource, providing a range of ecosystem services, including carbon storage, water, and pollutant regulation [33]. However, they are also vulnerable to disturbance, with activities such as drainage, mechanical extraction, conversion to other land uses and burning leading to the reduction of their resilience and the potential reduction of the ecosystem services enhancement [33].

The industrial cutaway bogs originated from a previously raised bog that was subjected to substantial peat extraction, predominantly to generate electricity in a condensing power facility. Although the extraction operations in these bogs have ceased, the substantial depletion of peat has been caused by previous activities. The forest plantation consists solely of Sitka spruce (aged between 12 and 15 years), adhering to the current Irish forestry best management practices (Forest Service, 2000). The management practices for the improved grassland involve frequent ploughing, regular re-seeding twice a year, and the application of slurry fertiliser at a rate of 40 kg/acre once a year. The stocking rate is relatively high, exceeding 3.3 cattle per hectare, and lime is regularly applied at a rate of 7.5 t/ha approximately every 2.5 years. In contrast, semi-natural grassland experiences little to no ploughing and almost no re-seeding in the past decade. Fertiliser application, including slurry, has been absent for the past ten years, with a low to medium stocking rate of less than 2.5 cattle per hectare. Lime application is limited, with less than 3.5 t/ha applied after three years.

### 2.2 Peatsoil sampling

In November 2021, a total of 16 samples of peatsoil were collected at 0–20 cm depths from various land use types including forestry, improved grassland, semi-natural grassland, and industrial cutaway. For each of these four types of peatland use, four plots were established, each measuring approximately 100 m × 100 m. Within each plot, composite samples of peatsoil were obtained by collecting ten cores from ten quadrants positioned at random distances along a diagonal line with dimensions of 0.5 m × 0.5 m. These samples were then sealed in plastic bags. Afterwards, the peatsoil samples were subjected to oven-drying at a temperature of 45 °C in the laboratory. They were then crushed and passed through a sieve with a 2-mm mesh size before being analysed for their soil chemical properties.

### 2.3 Soil characterisation and extraction of total heavy metal contents

The soil pH was measured in a 1 : 2.5 ratio of soil to water suspension using a glass electrode of a pH metre (Sparks et al., 2020). The loss of ignition method was employed to determine the soil organic carbon (SOC) (Agus et al., 2011). The microwave digestion extraction method was used for the determination of the selected heavy metals (Cu, Zn, Pb, Cd, Hg) and the other peatsoil properties (Mg, Ca, K and P) as described by the U.S. Environmental Protection Agency, [34]. Briefly, 0.2500 g of the sieved peatsoil was microwave digested with aqua regia extraction (10 mL concentrated nitric acid and 2 mL hydrochloric acid) at 120 °C for 45 min in the microwave digestor (MARS 6). The samples were filtered through a 0.45 mL membrane filter, transferred, and made up to a volume of 50 mL in a volumetric flask with ultrapure water before being analysed using inductively coupled plasma optical emission spectroscopy (ICP-OES) (Agilent 5800 ICP-OES) for the analysis of Mg, Ca, K, Cu, Zn, Pb and Cd while the ICP-MS (8900 Triple Quadrupole ICP-MS) was used to analysed the Hg. To ensure the accuracy of our measurements, we implemented quality control procedures that involved conducting replicated measurements, using blanks, and employing standard solutions. The accuracy of ICP-OES measurement was validated through the analysis of certified reference material (CRM) WEPAL-IPE-111. The recovery percentages for Cd, Cu Zn, and Hg, are 94.80 %, 92.32 %, 90.55 %, and 83.33 %, respectively (Table 1S). The detection limits for the studied elements in the ICP-OES measurements were as follows (in μg L^−1^): Mg = 2.25, Ca = 1.25, Pb = 6.30, Zn = 0.87, K = 0.21, P = 1.5, Cd = 0.32. The study limits for Hg was 0.6 μg L^−1^ in the ICP-MS measurements.

### 2.4 Methods of heavy metal contamination assessment

#### 2.4.1 Geo-accumulation index

The geo-accumulation index (Igeo) is a widely used method for assessing the contamination levels of heavy metals in soil pollution [38]. This index has been used in various studies to assess the pollution levels of heavy metals in different environmental settings, such as paddy fields [38], coastal municipalities [39], river sediments [40], surface water [41], urban soil [42] and agricultural soil [43]. It can be estimated using the following equation (1):

(1) $$Igeo=\log_{2}\left(\frac{Cs}{K*Bn}\right)$$

In the given equation, B_n_ represents the background value of the specific element in uncontaminated soil, C_s_ denotes the heavy metal concentration in peatsoils (mg kg^−1^), and K serves as a variation conversion factor, typically assigned a value of 1.5. This factor helps mitigate variations in background values that may arise from differences in geological compositions. In this study, the average world soil values for the heavy metals of uncontaminated soil under investigation were adopted as their background values. These values, as reported by Ref. [44] are 0.2, 27, 38.5, 0.05 and 70 mg kg^−1^ for Cd, Pb, Cu, Hg and Zn, respectively. For Cd, Pb, Cu, Hg, and Zn, the mean Igeo values offer a measure of the degree of contamination in each land type. These values can be interpreted as follows: for a given heavy metal if *I*_*geo*_≤0, the peatsoil is considered unpolluted, 0<*I*_*geo*_≤1, it is considered unpolluted to moderately polluted, 1<*I*_*geo*_≤2, moderately polluted, 2<*I*_*geo*_≤3, moderately to heavily polluted, 3<*I*_*geo*_≤4, heavily polluted, 4<*I*_*geo*_≤5, heavily to extremely polluted, *I*_*geo*_ > 5, extremely polluted [45].

#### 2.4.2 Soil contamination factor

The soil contamination factor often denoted as CF, is a parameter used to assess the degree of contamination in a given soil sample with respect to a specific heavy metal. It is estimated using the following equation (2):

(2) $$CF=\frac{Cs}{Bn}$$

where CF is the contamination factor of individual heavy metal, *B*_*n*_ represents the background value of the specific element, C_s_ denotes the heavy metal concentration in the peatsoils (mg kg^−1^). The interpretation of the soil contamination factor is based on the obtained value such that a CF less than 1 indicates low contamination, while a CF greater than 1 suggests varying degrees of contamination, with higher values representing more severe contamination.

#### 2.4.3 Hakanson potential ecological risk assessment

Hakanson's potential ecological risk (*RI*) assessment has been applied in various studies to evaluate the impact of heavy metal pollution on the environment [46,47]. both calculated toxicity coefficients for antimony and thallium, respectively, using Hakanson's principles. Fu et al. (2009) used the Hakanson method to assess the potential ecological risk of heavy metal pollution in sediments of Yangtze River in China [48]. Therefore, Hakanson's potential ecological risk (*RI*) was employed to assess the impact of heavy metal pollution of the studied peatland use type on the environment. The *RI* was calculated using the following equation (3):

(3) $$RI=\sum_{i=1}^{n}E_{r}^{i}=\sum_{i=1}^{n}T_{f}^{i}*CF=\sum_{i=1}^{n}T_{f}^{i}*\left(\frac{Cs}{Bn}\right)$$

where $E_{r}^{i}$ is the single metal potential ecological risk index for a given heavy metal, CF is the contamination factor of individual heavy metal and $T_{f}^{i}$ is toxic response factor and the toxic factor value for Cd, Pb, Cu, Hg and Zn used for the *R1* estimation were 30, 5, 5, 40 and 1, respectively [49,50]. The E_r_ (Ecological Risk) and *RI* (Risk Index) were categorized based on the classification scheme established by Hakanson. For $E_{r}^{i}$, the classification is as follows: $E_{r}^{i}$ <40 for low risk index; $E_{r}^{i}$ <40 for low risk index; 40≤ $E_{r}^{i}$ <80 for moderate risk index; 40≤ $E_{r}^{i}$ <80 for moderate risk index; 80≤ $E_{r}^{i}$ <160 for considerable risk index; 80≤ $E_{r}^{i}$ <160 for considerable risk index; 160≤ $E_{r}^{i}$ <320 for high risk index; 160≤ $E_{r}^{i}$ <320 for high risk index; $E_{r}^{i}$ ≥320 for very high risk index. $E_{r}^{i}$ ≥320 for very high-risk index. Similarly, for *RI*, the classification is as follows: *RI* < 95 for low-risk index; *RI* < 95 for low risk index; 95≤*RI* < 190 for moderate risk index; 95≤*RI* < 190 for moderate risk index; 190≤*RI* < 380 for considerable risk index; 190≤*RI* < 380 for considerable risk index; RI ≥ 380 for very high risk index; *RI* ≥ 380 for very high risk index.

### 2.5 Human health risk assessment

#### 2.5.1 Exposure assessment

In this study, an assessment of human health risks, encompassing both non-cancer and cancer risks, has been conducted for both adults and children (up to 12 years old). Soil heavy metals pose potential threats to human health through direct ingestion, inhalation through the mouth and nose and direct dermal contact. The calculation of average daily exposure doses (ADDs) for toxic metals across these diverse exposure pathways can be determined using the following (4), (5), (6) [51]:

(4) $$\text{ADDing}=C_{S}*\frac{{IR}_{ing}*EF*ED}{BW*AT}*10^{-6}$$

(5) $$\text{ADDinh}=C_{S}*\frac{{IR}_{inh}*EF*ED}{PEF*BW*AT}$$

(6) $$\text{ADDder}\;mal=C_{S}*\frac{SA*AF*ABS*EF*ED}{BW*AT}*10^{-6}$$

*ADD*_*ing*_, *ADD*_*inh*_ and ADD_dermal_ represent the cumulative daily intake resulting from direct ingestion, inhalation, and skin contact, respectively while the description and reference values for the parameters are presented in Table 1.

**Table 1:** Explanation and reference values of parameters used for the exposure's estimation.

Parameters	Age	Explanation	value	Ref
*IR*_ing_ (mg day^−1^)	children	Ingestion rate	200	[35]
	adult		100	
*R*_inh_ (m^3^ day^−1^)	children	Inhalation rate	7.5	[36]
	adult		14.5	
*ED (year)*	children	Exposure duration	6	[35]
	adult		24	
*SA* (cm^2^)	children	Exposed skin area	2800	[37]
	adult		5700	
*AF*(mg cm^−2^ day^−1^)	children	Skin adherence factor	0.07	[35]
	adult		0.2	
*BW (kg)*	children	Average bodyweight	15.9	[35]
	adult		56.8	
*ABS* (unitless)	Dermal absorption factor		0.001	[37]
*PEF* (m^3^ kg^−1^)	Particle emission factor		1.36*10^6^	[37]
*AT (day)*	Average exposure time		*ED* × 365	[35]
$C_{S\;}$ (mg kg ^−1^)	Heavy metal concentration			
*EF* (day year^−1^)	Exposure frequency			[35]

#### 2.5.2 Non-carcinogenic risk assessment

The assessment of non-carcinogenic risk involved calculating the Hazard Quotient (HQ) for each heavy metal and exposure pathway, along with the cumulative Hazard Index (HI). The HQs were determined individually for every identified heavy metal and corresponding exposure route. The evaluation of non-carcinogenic risk (HI) was conducted using equations (7), (8)) below [52]:

(7) $${HQ}_{ij}=\frac{{ADD}_{ij}}{{RfD}_{ij}}$$

(8) HI=∑HQ

where “i" and “j" indicate distinct types of heavy metals and specific exposure pathways, respectively. ${RfD}_{ij}$ represents the reference dose assigned to a particular heavy metal through a specified exposure pathway. The ingestion reference dose value for Hg, Pb, Cu, Zn and Cd are 3.00 × 10^−4,^ 3.50 × 10^−3^, 4.00 × 10^−2^, 3.00 × 10^−1^ and, 1.00 × 10^−3^ mg kg^−1^d^−1^, respectively; inhalation reference dose value for Hg, Pb, Cu, Zn and Cd are 8.57 × 10^−5^, 3.52 × 10^−3^, 4.02 × 10^−2^, 3.00 × 10^−1^ and 1.00 × 10^−3^ mg kg^−1^d^−1^, respectively and dermal contact reference dose value for Hg, Pb, Cu, Zn and Cd are 2.10 × 10^−5^, 5.25 × 10^−4^, 1.20 × 10^−2^, 6.00 × 10^−2^ and 1.00 × 10^−5^ mg kg^−1^d^−1^, respectively [53,54]. An HI value exceeding 1 suggests the potential for heavy metals to pose a non-carcinogenic risk. Conversely, an HI value below 1 indicates that the non-carcinogenic risk associated with heavy metals is not considered significant for the population under consideration [50].

#### 2.5.3 Carcinogenic risk assessment

Among the five studied heavy metals, Pb has been labelled as carcinogenic while Cd also holds carcinogenic properties [21,55]. As a result, our study concentrates on Cd and Pb as potential carcinogenic elements. The assessment of carcinogenic risk through ingestion was carried out using equations (9), (10)):

(9) $${CF=ADD}_{ij}*{SF}_{ij}$$

(10) $${CR=\sum CR}_{ij}$$

where “i" and “j" indicate distinct types of heavy metals and specific exposure pathways, ${\text{SF}}_{\text{ij}}$ represents the carcinogenic slope factor associated with a particular heavy metal through a specific route of exposure, CF is the contamination factor of individual heavy metal concentration, CR denotes the carcinogenic risk. The ingestion slope factor for Cd and Pb are 6.1 and 0.0085 mg kg^−1^d^−1^ respectively and inhalation slope factor for Cd are 6.3 mg kg^−1^d^−1^.

### 2.6 Data analysis

The data from the study were subjected to statistical analysis using one-way ANOVA in GraphPad Prism 10.12. A post-hoc analysis, specifically the Tukey HSD test, was conducted with a significance level (α) of 0.05 to determine the statistical significance of the data across different peatland use types, including improved and semi-natural grassland, industrial cutaway bog, and forest plantation. The interrelationships between the heavy metals and other selected soil chemical properties were assessed using correlation and Principal Component Analysis (PCA).

## 3 Results and discussion

### 3.1 Soil pH and SOC (%)

The soil pH varies significantly (*P* < 0.05) among the peatland use types (Table 2). The pH ranged from 4.74 to 6.22 with the lowest and the highest end of the ranges exhibited by forest and the improved grassland (Table 2). The pH levels in both the forest and the industrial cutaway showed no significant difference (*P* > 0.05), similar to the observations made between improved and semi-natural grassland areas (Table 2). The lowest pH exhibited by the forest can be attributed to the high concentration of hydrogen ions released during the decomposition of organic matter, particularly litterfalls build-up on the peatsoil that contain lignin [7,56]. Management practices, such as regular lime and fertiliser applications like manure and NPK fertiliser, explain the improved grassland's higher pH compared to the forest and industrial cutaway areas [56], such that this grassland management neutralises soil acidity and improves soil nutrients, promoting grass growth and supporting a different range of plant species than the forest and industrial cutaway areas [57,58].

**Table 2:** soil pH and SOC (%) of peatsoil affected by land use changes.

Peatland use types	Soil pH	Soil organic carbon (%)
Forest	4.74 ± 0.17^b^	48.58 ± 0.49^b^
Improved grassland	6.22 ± 0.32^a^	42.09 ± 1.57^c^
Semi-natural grassland	5.68 ± 0.15^a^	48.43 ± 0.80^b^
Industrial cutaway	4.84 ± 0.69^b^	54.59 ± 0.90^a^
Significant ^*X*^	***	***
F^*Y*^	12.89	87.91

Significant^*X*^ and F^*Y*^ effects were obtained from a one-way analysis of variance. Means followed by the same letter in each column are not significantly different at *p ≤ 0.05; **p ≤ 0.01***p ≤ 0.001 using Tukey.

The industrial cutaway showed a significantly higher (*P* < 0.05) SOC compared to the different types of peatland use that were studied (Table 2). This phenomenon occurs as a result of peat extraction, which exposes deeper layers of older, more decomposed peat with higher SOC that has accumulated over time [56]. No statistically significant differences (*P* > 0.05) were observed between the semi-natural grassland and the forest (Table 2). Meanwhile, the improved grassland had the lowest SOC levels of all the peatland uses studied (Table 2), most likely due to intensive management practices such as grazing, fertiliser application, and ploughing, which disrupted the natural balance of organic inputs and outputs within the improved grassland system [56,59,60].

### 3.2 Mg, ca, K, Zn, and P contents of peatsoil

**Fig. 2:**
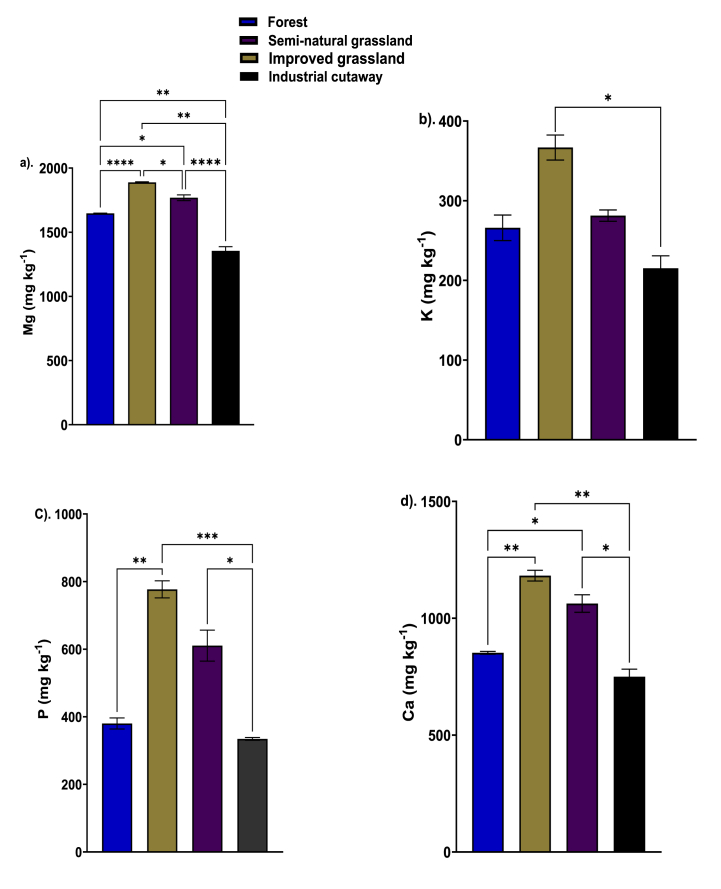
Effect of peatland use types on the content of Mg (a), K (b), P (c), and Ca (d) in forest, semi-natural grassland, improved grassland and industrial cutaway bog peatsoils. Significant differences are indicated by *p ≤ 0.05; **p ≤ 0.01; ***p ≤ 0.001; ****p ≤ 0.0001, as determined using Tukey HSD at the 0.05 significance level (n = 4).

The magnesium content differs significantly among the studied peatland use types (Fig. 2a). The magnesium content ranged from 1354.31 mg kg^−1^ to 1883.24 mg kg^−1^ with the lowest and the highest end of the range exhibited by the industrial cutaway bog and the improved grassland, respectively. The improved grassland showed a significantly higher magnesium content (*P* < 0.0001) compared to the forest but did not vary significantly when compared to that of the semi-natural grassland (Fig. 2a). The potassium content ranged from 215.16 mg kg^−1^ to 366.748 mg kg^−1^ with the lowest and the highest end of the ranges observed by the industrial cutaway and the improved grassland, respectively. The potassium content did not vary significantly among the forest, improved grassland, and semi-natural grassland. However, a significant difference (*P* < 0.05) was observed between the potassium content of the forest and the industrial cutaway (Fig. 2b). Grassland (both improved and semi-natural grassland) exhibited significant (*P* < 0.05) higher phosphorus content as compared to the phosphorus content observed by forestry and the industrial cutaway. The phosphorus content in the peatsoil from the forestry and the industrial cutaway bog did not vary significantly (*P* > 0.05) (Fig. 2c). The improved grassland had the greatest calcium content (1182.25 mg kg^−1^), followed by the semi-natural grassland (1063.1 mg kg^−1^), forestry (852.27 mg kg^−1^) and the industrial cutaway bog (749.92 mg kg^−1^) (Fig. 2d).

In comparing the selected chemical properties (Ca, Mg, K, Zn and P) of improved grassland with semi-natural grassland, forest peatsoil, and industrial cutaway bog, it becomes evident that the management practices (fertilizer application, tillage etc) applied to the improved grassland contribute significantly to the observed higher concentrations of Mg, Ca, K, Zn, and P [61,62]. For instance, the application of organic manure can increase the available P, K, and organic C content of the soil due to its high nutrient constituents (K, Ca, and Mg) [63,64]. Improved grassland management is likely to improve nutrient retention and utilisation, resulting in the observed increase in Mg, Ca, K, Zn, and P compared to semi-natural grassland, forest peatland, and industrial cutaway bog.

Additionally, the literature emphasizes the importance of soil-plant interactions and microbial activities in influencing nutrient dynamics in peatland ecosystems [65,66]. The improved grassland may exhibit a more favorable microbial community structure and activity due to high pH as a result of the manure and lime application, enhancing nutrient mineralization and cycling.

The differences in the Mg, Ca, K, Zn, and P contents between forested peatland and industrial cutaway bog can be attributed to the contrasting peatland use histories and vegetation dynamics of these two ecosystems. Forestry peatlands that have experienced relatively undisturbed natural processes and sustained plant growth tend to accumulate and retain higher concentrations of nutrients via enhanced aerobic peat mineralization (organic matter decomposition) and plant-microorganism interactions [66]. The enrichment is especially significant for nutrients such as Mg, Ca, and K, which are crucial for plant growth and microbial activity. The results presented in this study are consistent with those of Nieminen et al. [67] which showed high nutrient concentrations, specifically phosphorus, are caused by maturing tree stands and enhanced aerobic peat mineralization in forested peatlands following drainage stand growth and peat mineralization.

The lowest concentrations of Mg, Ca, K, Zn, and P exhibited by the industrial cutaway bogs can be assigned to the extensive peat extraction and disturbance that occurred in these areas, which frequently disrupts natural nutrient cycling processes and consequently reduces nutrient availability. The removal of vegetation and alteration of hydrological regimes contribute to reduced nutrient retention and increased leaching, resulting in lower concentrations of essential elements like Mg, Ca, K, Zn, and P [[68], [69]].

### 3.3 Heavy metal contents in peatsoil

**Fig. 3:**
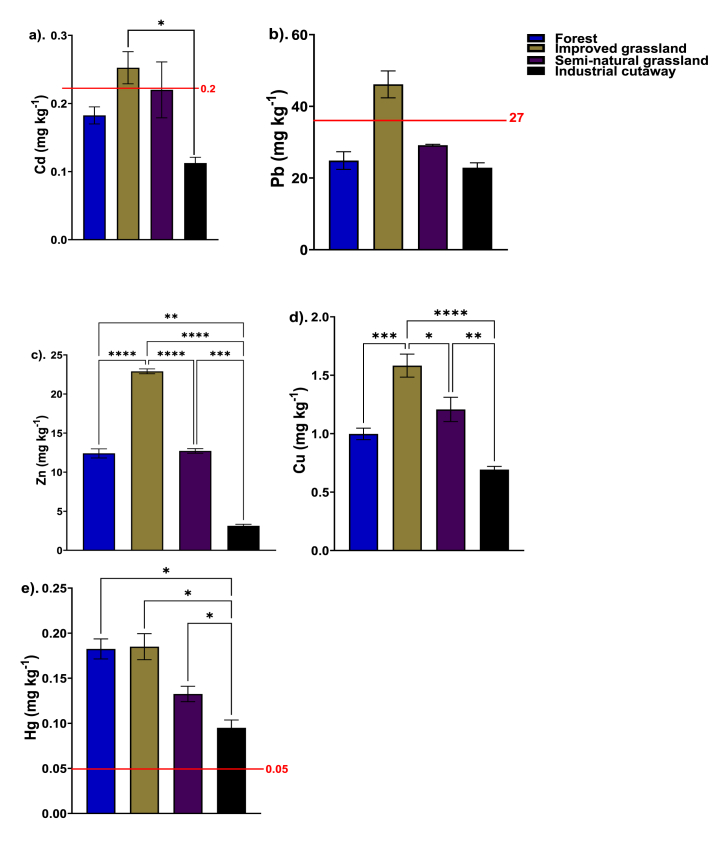
Effect of peatland use types on heavy metals content: Cd (a), Pb (b), Zn (c), Cu (d), and Hg (e) in forest, semi-natural grassland, improved grassland and industrial cutaway bog peatsoils. Significant differences are indicated by *p ≤ 0.05; **p ≤ 0.01; ***p ≤ 0.001; ****p ≤ 0.0001, as determined using Tukey HSD at the 0.05 significance level. The additional line denotes the global soil average or the baseline value (n = 4).

The improved grassland had the greatest Cd content (0.841 mg kg^−1^), followed by the semi-natural grassland (0.7333 mg kg^−1^), forestry (0.608 mg kg^−1^) and the industrial cutaway bog (0.375 mg kg^−1^) (Fig. 3a). The Pb did not vary significantly (*P* > 0.05) among the peatland use types, with measurements of improved grassland (46.13 mg kg^−1^) > semi-natural grassland (29.16 mg kg^−1^), forestry (24.88 mg kg^−1^) and Industrial cutaway (22.87 mg kg^−1^) (Fig. 3b). The concentration of Cd and Pb in the grassland peatsoils (both, improved and semi-natural) was higher and found to exceed the WHO permissible safety limits. These concentrations exceed the levels found in forested peatland and industrial cutaway bog. The grassland especially the improved grassland, was subjected to consistent fertilisation with cow dung manure to enhance soil fertility which may likely be responsible for the increased concentrations of trace metals detected in the peatsoil of the grassland. Previous study shown that heavy metals accumulated in the soil can be influenced significantly by fertilisers and pesticide use [30] which may account for the observed difference in heavy metal levels between grassland and forested peatlands.

The Zn content varies significantly (*P* < 0.05) among the peatland use types. The Zn content ranged from 3.13 to 22.91 mg kg^−1^ with the lowest and the highest end of the ranges recorded by improved grassland and the industrial cutaway, respectively (Fig. 3c). The improved grassland significantly (*P* < 0.05) measured higher Zn content than the forestry, semi-natural grassland and industrial cutaway (Fig. 3c). The Zn content among the forestry and the semi-natural grassland did not differ significantly (*P* > 0.05) (Fig. 3c). The Cu content varies significantly (*P* < 0.005) among the peatland use types (Fig. 3d). The Cu contents ranged from 0.692 mg kg^−1^ to 1.583 mg kg^−1^ with the lowest and the highest Cu exhibited by the improved grassland and the industrial cutaway (Fig. 3d). The improved grassland showed a significantly higher Cu content than the semi-natural grassland, forest, and industrial cutaway (Fig. 3d). Meanwhile, the Hg ranged from 0.095 mg kg^−1^ to 0.183 mg kg^−1^ with the lowest and the highest Hg content observed by the industrial cutaway and the improved grassland, respectively (Fig. 3e). The Hg content in the peatsoil from the grassland and the forest did not vary significantly (*P* > 0.05) (Fig. 3e). The Cu, Zn and Hg were higher in the grassland (both the improved and semi-natural) than forest peatsoils and industrial cutaway bog which were below the WHO permissible safety limits excerption of the Hg.

The availability of heavy metals in peatsoil is influenced by a combination of factors, with soil pH and organic matter content playing significant roles. Studies have shown that the interaction between these two factors can have a stronger influence on heavy metal availability than either factor alone [70]. The presence of higher organic matter can bind heavy metals, reducing their availability [71], while the pH of the peatsoil can affect the solubility and mobility of these metals [72]. In this study, we demonstrated distinct levels of soil organic carbon (SOC) and pH across various peatland use types (Table 2). Therefore, this contrast in SOC and pH levels between grasslands, along with their varying degrees, offers insights into the high heavy metal concentrations observed in these areas compared to forest peatlands and industrial cutaway bogs.

The average contents in all the peatsoils were in the order Pb > Zn > Cu > Cd > Hg. The presence of heavy metals in peatsoils is a common phenomenon, with studies reporting high concentrations, high altitude peat bogs [73], and peat bogs used for historical monitoring of trace metals [74] in which the enrichment of these trace metals in peat profiles has been attributed to both natural and anthropogenic factors [75], with the latter being linked to human settlement and industrial activities [76].

### 3.4 Geo-accumulation index of heavy metals in peatsoil

The geo-accumulation index (Table 3) shows the mean Igeo values of Cd, Pb, Cu, Hg and Zn for forestry, improved grassland, semi-natural grassland and industrial cutaway, showing negative values for Cu, Hg, Cd and Zn. The mean Igeo of Pb ranged from −0.832 to 0.173, with the industrial cutaway and improved grassland exhibiting the lowest and highest ends, respectively (Table 3). The Igeo values for Cd, Cu, Hg, and Zn consistently demonstrate relatively negative values across the studied peatland use types, indicating an absence of significant contamination with these metals [77]. This suggests that these metals are more likely of natural origin rather than being anthropogenically introduced [78]. However, a noteworthy contrast is observed in the case of Pb, where the Igeo values for improved grassland peatsoils are positive and less than one. This signifies a higher potential for Pb contamination in the future, implying a need for closer monitoring and potential intervention measures in the improved grassland areas to prevent or mitigate further contamination.

**Table 3:** Geo-accumulation index of potentially toxic elements in forest, grassland (Semi-natural and improved) and Industrial cutaway bog.

Peatland use types	Cd	Pb	Cu	Hg	Zn
Forestry	−0.728 ± 0.204^ab^	−0.723 ± 0.272^b^	−5.864 ± 0.142^b^	−2.510 ± 0.192^b^	−3.086 ± 0.136^b^
Improved grassland	−0.266 ± 0.256^a^	0.173 ± 0.234^a^	−5.199 ± 0.190^a^	−2.047 ± 0.174^a^	−2.197 ± 0.039^a^
Semi-natural grassland	−0.532 ± 0.588^a^	−0.474 ± 0.025^b^	−5.591 ± 0.117^b^	−2.032 ± 0.228^a^	−3.047 ± 0.066^b^
Industrial Cutaway	−1.428 ± 0.228^b^	−0.832 ± 0.179^b^	−6.386 ± 0.120^c^	−2.999 ± 0.265^c^	−5.076 ± 0.188^c^
Significant^X^	***	***	***	***	***
F^Y^	7.833	20.169	31.677	17.735	37.359

Significant^*X*^ and F^*Y*^ effects were obtained from a one-way analysis of variance. Mean ± SD followed by the same letter in each column are not significantly different at *p ≤ 0.05; **p ≤ 0.01***p ≤ 0.001 using Tukey HSD at the 0.05 significance level. (n = 3).

### 3.5 Soil contamination factor

**Fig. 4:**
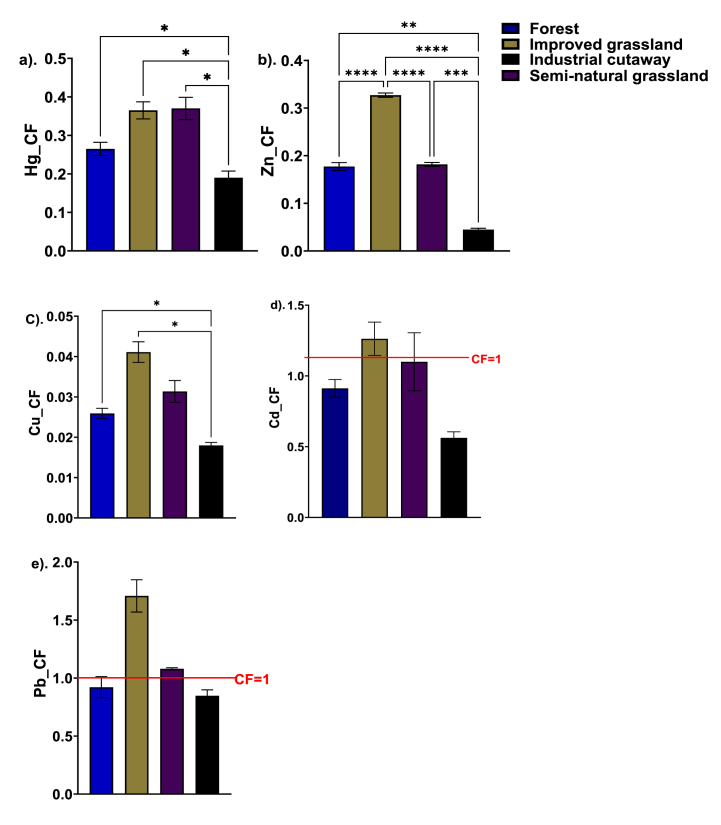
Soil contamination factor of Hg (a), Zn (b), Cu (c), Cd (d), and Pb (e) in different soil types: forest, semi-natural grassland, improved grassland, and industrial cutaway peatsoils. Significant differences are indicated by *p ≤ 0.05; **p ≤ 0.01; ***p ≤ 0.001; ****p ≤ 0.0001, as determined using Tukey HSD at the 0.05 significance level.

In this study, the CF for the Hg (Fig. 4a), Zn (Fig. 4b), Cu (Fig. 4c), Cd (Fig. 4d) and Pb (Fig. 4e) were below one across the studied peatland use type, with the exception of the CF for Cd (Fig. 4d) and Pb (Fig. 4e) in the grassland peatsoil (both the improved and semi-natural). The CF for Cd and Pb exceeding one may be influenced by management practices such as fertilizer and pesticide use, as well as atmospheric deposition [74,79]. In contrast, the forest peatland may exhibit lower contamination factors due to effective land management practices and natural protective mechanisms [80]. The presence of dense vegetation cover and natural processes in forest peatlands may contribute to the immobilization or reduced mobility of Cd and Pb, resulting in lower contamination factors [81].

### 3.6 Hakanson potential ecological risk assessment

**Fig. 5:**
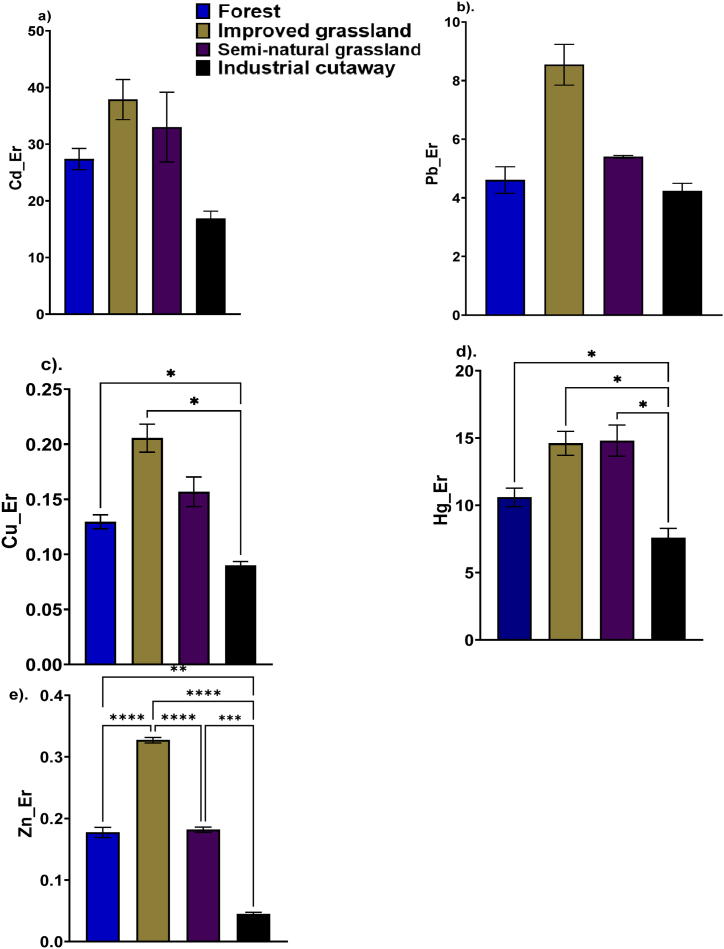
Single metal potential ecological risk factor of Cd (a), Pb (b), Cu (c), Hg (d), and Zn (e) in different peatsoil types: forest, semi-natural grassland, improved grassland, and industrial cutaway peatsoils. Significant differences are indicated by *p ≤ 0.05; **p ≤ 0.01; ***p ≤ 0.001; ****p ≤ 0.0001, as determined using Tukey HSD at the 0.05 significance level. in forest, grassland (Semi-natural and improved) and Industrial cutaway peatsoil.

The Er for Cd did not differ significantly between the semi-natural grassland, forestry and the industrial cutaway bog. The improved grassland had the greatest Er for Cd followed by the semi-natural grassland (33.12), forestry (27.23) and the industrial cutaway bog (16.87) (Fig. 5a). The Er for Pb did not vary significantly (*P* > 0.05) among the peatland use types and was of the order improved grassland (8.542) > semi-natural grassland (5.40), forestry (4.61) and Industrial cutaway (4.24) (Fig. 5b). The Er for Cu differed significantly (*P* < 0.05) among the peatland use types and ranged from 0.899 to 0.255 with the lowest and the highest exhibited by the improved grassland and the industrial cutaway (Fig. 5c). The Er for Hg ranged from 7.6 to 14.8 with the lowest and the highest values for industrial cutaway and improved grassland, respectively (Fig. 5d). The values for grassland (both improved and semi-natural) and the forest did not vary significantly (*P* > 0.05) (Fig. 5d). The Er for Zn varies significantly (*P* < 0.05) among the peatland use types (Fig. 5e) and ranged from 0.045 for improved grassland to 0.33 for industrial cutaway (Fig. 5e). The improved grassland was significantly (*P* < 0.05) higher than the forestry, semi-natural grassland and industrial cutaway, while the forestry and the semi-natural grassland did not differ significantly (*P* > 0.05) (Fig. 5e).

The studied heavy metals within the studied peatland use types suggested a generally low contamination level based on the Hakanson potential ecological risk (Er) values. The calculated values for Cu, Hg, Pb, Zn, and even Cu fell within a range indicative of minimal ecological risk. However, an exception was observed with Cd, which demonstrated an Er value of 37.9 in improved grassland peatsoils. Therefore, this finding raises concerns about the potential for Cd to transition from a low to a moderate ecological risk in the near future. Several studies revealed a moderate to high ecological risk associated with Cd in various ecosystems [[82], [83], [84], [85], [86]]. For instance, Zhou et al. [86], found a high potential ecological risk in farmland soils in China, with Cd, Pb, and Zn being heavily polluted. Rostami et al. [83], found low to moderate contamination and risk in agricultural soils in Iran, with Cd posing the highest risk. Tang et al. [84], identified slight to intense potential ecological risk in soils in China, with Cd and Pb being the main contributors. Xu et al. [85], found heavy contamination and high potential ecological risk in paddy soils from Pb–Zn mining areas in China, with Cd and Pb posing the highest risk. The high Er for Cd in the improved grassland peatsoils imply a heightened likelihood of environmental impact, necessitating a closer examination of specific management practices contributing to the increased ecological risk associated with this in the improved grassland peatsoils.

**Fig. 6:**
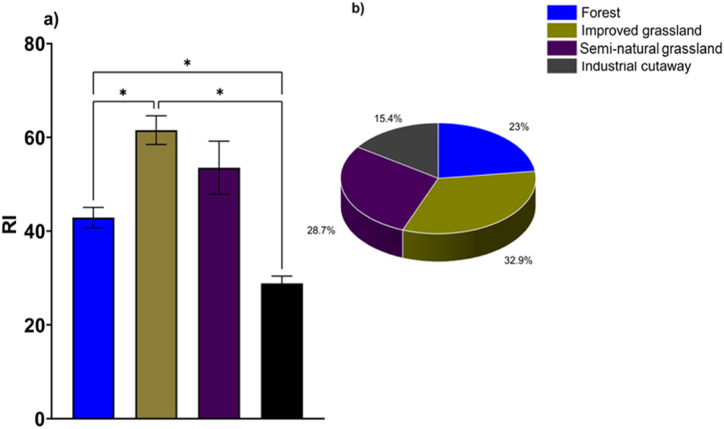
The potential ecological risk indexes (*RI*) (a) and the percentage distribution of potential ecological risk index (b) of the four peatland use types. *p ≤ 0.05; **p ≤ 0.01***p ≤ 0.001; ****p ≤ 0.0001 using Tukey HSD at the 0.05 significance level.

The *RI* differs significantly (*P* < 0.05) among the peatland use types. The range of *RI* values varied from 28.85 to 61.55, with industrial cutaway having the lowest value and improved grassland the highest. Specifically, semi-natural grassland exhibited an *RI* of 53.54, which was not significantly different from the forest (42.88) as indicated in Fig. 6a. However, both semi-natural grassland and forest differed significantly from industrial cutaway (Fig. 6a), where the *RI* was at its lowest. The percentage distribution of potential ecological risk index of the four peatland-use types followed the trend: improved grassland (32.9 %) > semi-natural grassland (28.7 %) > forest (23) > industrial cutaway (15.5 %) (Fig. 6b). The *RI* values for the five heavy metals in all four peatland use types were below 150, suggesting a low ecological risk associated with these heavy metals.

### 3.7 Human health risk assessment

#### 3.7.1 Exposure assessment

The examined heavy metals present in the soil can potentially enter the human body through three primary exposure pathways: hand-to-mouth ingestion, respiratory inhalation, and dermal contact. Among these pathways, hand-to-mouth ingestion emerged as the predominant risk route across all four investigated peatland use types. This pathway accounted for over 99 % of the total Average Daily Dose (ADD) in both adults and children, as indicated in Table 4. The total Average Daily Dose (ADD) of exposure to all five heavy metals for children was higher across the different peatland types: forestry (4.64E-04 mg kg^−1^ day^−1^), improved grassland (8.56E-04 mg kg^−1^ day^−1^), semi-natural grassland (5.23E-04 mg kg^−1^ day^−1^) and industrial cutaway (3.24E-04 mg kg^−1^ day^−1^) compared to that for adults across the same peatland types: forestry (2.60E-04 mg kg^−1^ day^−1^), improved grassland (4.80E-04 mg kg^−1^ day^−1^), semi-natural grassland (2.93E-04 mg kg^−1^ day^−1^) and industrial cutaway (1.87E-04 mg kg^−1^ day^−1^) (Table S2). Numerous studies have consistently highlighted the ingestion pathway as the primary exposure route, accounting for 97 % of the total risk value for heavy metal contamination in soil, with significant implications for human health [22,29,87,88].

**Table 4:** Ratio of each average daily dose (ADD) via three exposure routes to total ADD for adults and children.

Peatland use types	Group	Hand–mouth ingestion %	Dermal contact (%)	Respiratory inhalation (%)
Forestry	Adult	99.811	0.179	0.106
	Children	99.912	0.086	0.003
Improved grassland	Adult	99.811	0.179	0.011
	Children	99.912	0.086	0.003
Semi-natural grassland	Adult	99.810	0.179	0.011
	Children	99.911	0.086	0.003
Industrial cutaway	Adult	99.816	0.173	0.010
	Children	99.911	0.087	0.003

#### 3.7.2 Non-carcinogenic risk assessment

The values of Hazard Quotient (HQ) and Hazard Index (HI) for the five heavy metals across different peatland use types are summarized in Table 5. The Cd HI values for children ranged from 1.358E-03 to 3.049E-03 with the lowest and the highest end of the range exhibited by the industrial cutaway bog and the improved grassland, respectively. Similar trends were observed for Pb, Cu, Hg and Zn as the industrial cutaway bog and the improved grassland exhibited the lowest and highest. The Hazard Index (HI) values for children exhibited a descending order: Zn > Hg > Cu > Cd > Pb, consistently observed across the four peatland use types. Similarly, for adults, this order (Zn > Hg > Cu > Cd > Pb) persisted across forestry, improved grassland, semi-natural grassland, and industrial cutaway. The risk assessment indicated no non-carcinogenic risk for either children or adults based on individual heavy metals (HQ < 1 for each heavy metal) within the studied peatland types, as detailed in Table 5. The non-carcinogenic risk, as indicated by HQ values below 1 for each heavy metal (Cd, Pb, Cu, Hg and Zn) and the combined exposure to all heavy metals, as reflected in the Hazard Index (HI), consistently fell below the safety level (HI < 1). This implies that both individually and in combination, the concentrations of Zn, Hg, Cu, Cd, and Pb in the studied peatland areas do not pose a significant non-carcinogenic risk to either children or adults. The absence of non-carcinogenic risk in the studied peatland areas is consistent with findings in other regions, where heavy metal concentrations such as Zn, Hg, Cu, Cd, and Pb also did not pose significant non-carcinogenic risks [18,89,90]. However, this is in contrast to studies in South China, the northeastern Qinghai-Tibet Plateau, and urban soil in China, where Cu, Cd, and Pb pollution and associated health risks were found to be significant [91,92].

**Table 5:** Non-carcinogenic risk of different heavy metals to adults and children under different peatland use.

Peatland use type	Age	Cd	Pb	Cu	Hg	Zn	Total ADD
**HQ**_**ing**_							
Forestry	Adult	1.232E-03	4.799E-02	1.684E-04	2.983E-04	2.791E-04	4.997E-02
	Children	2.201E-03	8.572E-02	3.008E-04	5.327E-04	4.985E-04	8.926E-02
Improved grassland	Adult	1.705E-03	8.899E-02	2.672E-04	4.108E-04	5.155E-04	9.189E-02
	Children	3.046E-03	1.590E-01	4.772E-04	7.338E-04	9.207E-04	1.642E-01
Semi-natural grassland	Adult	1.486E-03	5.627E-02	2.039E-04	4.164E-04	2.863E-04	5.866E-02
	Children	2.654E-03	1.005E-01	3.641E-04	7.438E-04	5.113E-04	1.048E-01
Industrial Cutaway	Adult	7.597E-04	4.413E-02	1.169E-04	2.138E-04	7.051E-05	4.530E-02
	Children	1.357E-03	7.883E-02	2.088E-04	3.820E-04	1.259E-04	8.091E-02
**HQ**_**dermal**_							
Forestry	Adult	1.314E-07	5.088E-06	1.786E-08	1.113E-07	1.488E-07	5.497E-06
	Children	6.070E-08	2.350E-06	8.253E-09	5.142E-08	6.873E-08	2.539E-06
Improved grassland	Adult	1.818E-07	9.434E-06	2.834E-08	1.533E-07	2.748E-07	1.007E-05
	Children	8.398E-08	4.358E-06	1.309E-08	7.082E-08	1.269E-07	4.653E-06
Semi-natural grassland	Adult	1.584E-07	5.965E-06	2.163E-08	1.554E-07	1.526E-07	6.453E-06
	Children	7.317E-08	2.755E-06	9.990E-09	7.179E-08	7.049E-08	2.980E-06
Industrial Cutaway	Adult	8.100E-08	4.679E-06	1.240E-08	7.981E-08	3.759E-08	4.890E-06
	Children	3.742E-08	2.161E-06	5.729E-09	3.687E-08	1.736E-08	2.259E-06
**HQ**_**inh**_							
Forestry	Adult	2.207E-06	8.596E-05	3.016E-07	7.632E-06	2.500E-06	9.860E-05
	Children	1.886E-06	7.345E-05	2.577E-07	6.521E-06	2.136E-06	8.425E-05
Improved grassland	Adult	3.054E-06	1.594E-04	4.785E-07	1.051E-05	4.616E-06	1.781E-04
	Children	2.609E-06	1.362E-04	4.089E-07	8.981E-06	3.944E-06	1.521E-04
Semi-natural grassland	Adult	2.661E-06	1.008E-04	3.651E-07	1.066E-05	2.564E-06	1.171E-04
	Children	2.274E-06	8.611E-05	3.120E-07	9.104E-06	2.190E-06	9.999E-05
Industrial Cutaway	Adult	1.361E-06	7.905E-05	2.094E-07	5.472E-06	6.315E-07	8.672E-05
	Children	1.163E-06	6.754E-05	1.789E-07	4.675E-06	5.395E-07	7.410E-05
**HI**							**Total HI**
Forestry	Adult	1.234E-03	4.808E-02	1.687E-04	3.060E-04	2.817E-04	5.007E-02
	Children	2.203E-03	8.580E-02	3.011E-04	5.393E-04	5.007E-04	8.934E-02
Improved grassland	Adult	1.708E-03	8.916E-02	2.677E-04	4.215E-04	5.204E-04	9.208E-02
	Children	3.049E-03	1.591E-01	4.776E-04	7.429E-04	9.248E-04	1.643E-01
Semi-natural grassland	Adult	1.489E-03	5.638E-02	2.043E-04	4.272E-04	2.890E-04	5.879E-02
	Children	2.656E-03	1.006E-01	3.644E-04	7.530E-04	5.136E-04	1.049E-01
Industrial Cutaway	Adult	7.611E-04	4.422E-02	1.171E-04	2.194E-04	7.118E-05	4.539E-02
	Children	1.358E-03	7.890E-02	2.090E-04	3.867E-04	1.265E-04	8.098E-02

#### 3.7.3 Carcinogenic risk assessment

Cd exposure via the dermal pathway remained below the acceptable level (1 × 10^−6^) across the forestry, improved grassland, semi-natural grassland and industrial cutaway (Table 6). On the other hand, exposure to Cd and Pb through the ingestion pathway poses potential carcinogenic risks, as their cancer risk (CR) levels exceeded the acceptable threshold (1 × 10^−6^) across the various peatland use types studied, impacting both children and adults (Table 6). This risk is especially evident in children, who consistently have a higher CR than adults across all peatland use types studied. This is because developing organ systems, hand-to-mouth activity, and higher rates of ingestion per unit of body weight among children render them more susceptible to heavy metal exposure [93]. The carcinogenic risk associated with Cd and Pb ingestion is due to their accumulation in the body, interference with DNA repair mechanisms, and mimicry of essential metals [94]. Cd can disrupt cell cycle progression, apoptosis, and DNA repair [21], while Pb can disrupt signalling pathways and cause inflammation [95]. Zinc and multi-mineral supplements reduce Cd's pathogenic effects [96]. Oxidative stress, DNA damage, and cell death make these metals toxic and carcinogenic [55]. Probiotic treatment may prevent Pb from impairing gut physiology and microbiota [95], while Cd can disrupt DNA damage response and cell growth [97]. The improved grassland demonstrated a higher CR than the other three peatland use types (forestry, semi-natural grassland, and industrial cutaway), indicating a heightened risk associated with Cd, which exhibited a higher CR than Pb.

**Table 6:** Carcinogenic risk of different heavy metals to adults and children under different peatland use.

Peatland use type	Age	Cd	Pb
CR_ing_			
Forestry	Adult	7.518E-06	1.428E-06
	Children	13.431E-06	2.550E-06
Improved grassland	Adult	1.040E-05	2.648E-06
	Children	1.858E-05	4.729E-06
Semi-natural grassland	Adult	9.062E-06	1.674E-06
	Children	1.619E-05	2.990E-06
Industrial Cutaway	Adult	4.634E-06	1.313E-06
	Children	8.277E-06	2.345E-06
**CR**_**dermal**_			
Forestry	Adult	8.278E-10	–
	Children	1.428E-06	–
Improved grassland	Adult	1.145E-09	–
	Children	5.291E-10	–
Semi-natural grassland	Adult	9.979E-10	–
	Children	4.610E-10	–
Industrial Cutaway	Adult	5.103E-10	–
	Children	2.357E-10	–
**CR**			
Forestry	Adult	7.518E-06	1.428E-06
	Children	1.486E-05	2.550E-06
Improved grassland	Adult	1.040E-05	2.648E-06
	Children	1.858E-05	4.729E-06
Semi-natural grassland	Adult	9.063E-06	1.674E-06
	Children	1.619E-05	2.990E-06
Industrial Cutaway	Adult	4.634E-06	1.313E-06
	Children	8.278E-06	2.345E-06

### 3.8 Principal component analysis (PCA) and correlation analysis

**Fig. 7:**
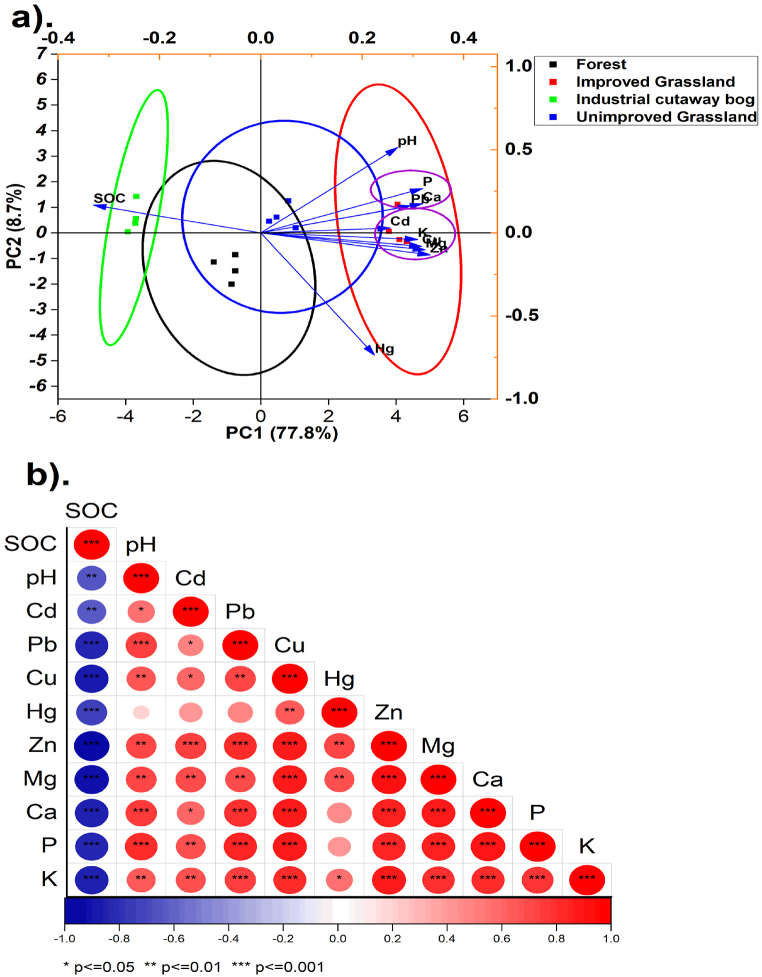
The principal component analysis (a) and Pearson's correlation analysis (b) of soil chemical properties under different peatland use.

Principal component analysis (PCA) and Pearson correlation were used to examine the relationship among the soil chemical properties. (Fig. 7). Two PCAs were extracted explaining 86.5 % of the overall variance. PCA 1, which was the most significant, explained 77. 8 % of the overall variance, with significant contributions from Cd, Pb, Cu, Hg, Zn, Mg, Ca, P, pH and K (Fig. 7a). The grouping of Cd, Cu, Zn, K and Mg at the negative loading implies that these elements may have common origins (Fig. 7a). Positive loading was observed for SOC which accounted for 8.72 % of the overall variance. By analyzing the spatial distribution of the points in the PCA, it can be noted that the peatsoil under the improved grassland is moving closer to the unimproved grassland condition, indicating some similarities in their soil chemical properties. The improved grassland and the industrial cutaway were separated along PCA 1. The improved grassland is associated with higher levels of Cd, Cu, Zn, K, and Mg, while the industrial cutaway is associated with higher levels of SOC.

Pearson's correlation analysis among the soil chemical parameters across different peatland use types (Fig. 7b) reveals statistically significant relationships. The SOC had a significant negative correlation with the studied heavy metals and other soil chemical parameters. SOC is required to bind heavy metals via complexation and adsorption processes, so as SOC increases, more organic functional groups become available for metal binding, reducing their bioavailability and mobility in soil [98]. The positive correlation between pH and the metals Cd, Pb, Cu, Hg, and Zn indicates that as pH increases, the solubility of these metals in soil may also increase due to changes in their speciation [10]. Zn, Mg, P, and K exhibit significant correlations with Cd, Cu, and Pb. The analysis indicates statistically significant correlations between Mg and Hg, implying a potential common source for these elements. Also, the correlations between Pb and Cd, as well as Pb and Cu, are found to be statistically significant. These findings highlight the interrelatedness of soil chemical concentrations in the soil across various peatland uses, indicating common origins and possible environmental interactions (Fig. 7b).

## 4 Conclusion

The findings of this comprehensive study provide insight into heavy metals concentration and the associated risks in peatlands under different land use types. Among the peatland use types, grassland (both improved and semi-natural) had the highest levels of Cd, Hg, and Pb, which exceeded WHO thresholds, highlighting the vulnerability of these areas to contamination due to management practices such as fertiliser application. The higher contamination factors (CF) in improved grassland, particularly for Cd and Pb, emphasize the potential ecological consequences of human activities in these regions. While the overall ecological risk levels, as assessed by Hakanson potential ecological risk, were considered acceptable, the variations observed among different peatland use types call for careful monitoring and management strategies. Further, the combined exposure to heavy metals falls below non-carcinogenic risk thresholds for both children and adults across peatland use types provides some reassurance. Nevertheless, the higher cancer risk, especially in improved grassland areas and for children, highlights the importance of considering long-term health implications associated with heavy metal exposure in these environments.

## CRediT authorship contribution statement

**Samuel Obeng Apori:** Writing – review & editing, Writing – original draft, Software, Methodology, Investigation, Formal analysis, Data curation, Conceptualization. **Michelle Giltrap:** Writing – review & editing, Visualization, Validation, Supervision, Software, Project administration, Methodology, Investigation, Formal analysis, Data curation, Conceptualization. **Julie Dunne:** Writing – review & editing, Validation, Supervision, Methodology. **Furong Tian:** Writing – review & editing, Writing – original draft, Visualization, Validation, Supervision, Resources, Project administration, Methodology, Investigation, Funding acquisition, Formal analysis, Data curation, Conceptualization.

## Declaration of competing interest

The authors declare that they have no known competing financial interests or personal relationships that could have appeared to influence the work reported in this paper.
