# Supplementary material for: Human health and ecological risk assessment of heavy metals in topsoil of different peatland use types
Source: Heliyon. 2024 Jun 25;10(13):e33624. doi: 10.1016/j.heliyon.2024.e33624 (PMC11260962; doi:10.1016/j.heliyon.2024.e33624)
Supplement: Multimedia component 1 [file mmc1.docx]

| Element | Certified Value  (mg/kg) | Measured value | Recovery  (%) |
| --- | --- | --- | --- |
| Cd | 0.481 | 0.456 | 94.80 |
| Zn | 38.1 | 34.5 | 90.55 |
| Cu | 5.74 | 5.30 | 92.33 |
| Hg | 0.012 | 0.01 | 83.33 |
| Ca | 16400 | 15891 | 96.90 |
| K | 22600 | 21420 | 94.78 |
| Mg | 1130 | 1013 | 89.65 |
| P | 3360 | 3090 | 91.96 |

**Table 1S. Measured and certified values of the metals in WEPAL-IPE-111**

**Table S2:** A**DD of each heavy metal through the three exposure routes for adults and children (mg kg^-1^ day^-1^)**

| **Peatland use type** | **Age** | **Cd** | **Pb** | **Cu** | **Hg** | **Zn** |
| --- | --- | --- | --- | --- | --- | --- |
| **ADD_ing_** |  |  |  |  |  |  |
| Forestry | Adult | 1.232E-06 | 1.680E-04 | 6.736E-06 | 8.948E-08 | 8.374E-05 |
|  | Children | 2.201E-06 | 3.000E-04 | 1.203E-05 | 1.598E-07 | 1.496E-04 |
| Improved grassland | Adult | 1.705E-06 | 3.115E-04 | 1.069E-05 | 1.232E-07 | 1.546E-04 |
|  | Children | 3.046E-06 | 5.563E-04 | 1.909E-05 | 2.201E-07 | 2.762E-04 |
| Unimproved grassland | Adult | 1.486E-06 | 1.969E-04 | 8.154E-06 | 1.249E-07 | 8.588E-05 |
|  | Children | 2.654E-06 | 3.517E-04 | 1.456E-05 | 2.231E-07 | 1.534E-04 |
| Industrial Cutaway | Adult | 7.597E-07 | 1.545E-04 | 4.676E-06 | 6.415E-08 | 2.115E-05 |
|  | Children | 1.357E-06 | 2.759E-04 | 8.353E-06 | 1.146E-07 | 3.778E-05 |
| **ADD_dermal_** |  |  |  |  |  |  |
| Forestry | Adult | 2.207E-09 | 3.009E-07 | 1.207E-08 | 1.603E-10 | 1.500E-07 |
|  | Children | 1.886E-09 | 2.571E-07 | 1.031E-08 | 1.369E-10 | 1.281E-07 |
| Improved grassland | Adult | 3.054E-09 | 5.579E-07 | 1.914E-08 | 2.207E-10 | 2.770E-07 |
|  | Children | 2.609E-09 | 4.767E-07 | 1.635E-08 | 1.886E-10 | 2.367E-07 |
| Unimproved grassland | Adult | 2.661E-09 | 3.527E-07 | 1.461E-08 | 2.238E-10 | 1.538E-07 |
|  | Children | 2.274E-09 | 3.014E-07 | 1.248E-08 | 1.912E-10 | 1.314E-07 |
| Industrial Cutaway | Adult | 1.361E-09 | 2.767E-07 | 8.376E-09 | 1.149E-10 | 3.789E-08 |
|  | Children | 1.163E-09 | 2.364E-07 | 0.716E-08 | 9.818E-11 | 3.237E-08 |
| **ADD_inh_** |  |  |  |  |  |  |
| Forestry | Adult | 1.314E-10 | 1.791E-08 | 7.182E-10 | 9.540E-12 | 8.928E-09 |
|  | Children | 6.070E-11 | 8.273E-09 | 3.318E-10 | 4.407E-12 | 4.124E-09 |
| Improved grassland | Adult | 1.818E-10 | 3.321E-08 | 1.139E-09 | 1.314E-11 | 1.649E-08 |
|  | Children | 8.398E-11 | 1.534E-08 | 5.263E-10 | 6.070E-12 | 7.616E-09 |
| Unimproved grassland | Adult | 1.584E-10 | 2.100E-08 | 8.694E-10 | 1.332E-11 | 9.156E-09 |
|  | Children | 7.317E-11 | 9.699E-09 | 4.016E-10 | 6.153E-12 | 4.230E-09 |
| Industrial Cutaway | Adult | 8.100E-11 | 1.647E-08 | 4.986E-10 | 6.840E-12 | 2.255E-09 |
|  | Children | 3.742E-11 | 7.608E-09 | 2.303E-10 | 3.160E-12 | 1.042E-09 |
| **Total ADD** |  |  |  |  |  |  |
| Forestry | Adult | 1.23E-06 | 1.68E-04 | 6.75E-06 | 8.96E-08 | 8.39E-05 |
|  | Children | 2.20E-06 | 3.00E-04 | 1.20E-05 | 1.60E-07 | 1.50E-04 |
| Improved grassland | Adult | 1.71E-06 | 3.12E-04 | 1.07E-05 | 1.23E-07 | 1.55E-04 |
|  | Children | 3.05E-06 | 5.57E-04 | 1.91E-05 | 2.20E-07 | 2.76E-04 |
| Unimproved grassland | Adult | 1.49E-06 | 1.97E-04 | 8.17E-06 | 1.25E-07 | 8.60E-05 |
|  | Children | 2.66E-06 | 3.52E-04 | 1.46E-05 | 2.23E-07 | 1.54E-04 |
| Industrial Cutaway | Adult | 6.60E-06 | 1.55E-04 | 4.68E-06 | 6.43E-08 | 2.12E-05 |
|  | Children | 1.36E-06 | 2.76E-04 | 8.36E-06 | 1.15E-07 | 3.78E-05 |
